# Primary progressive multiple sclerosis in a Russian cohort: relationship with gut bacterial diversity

**DOI:** 10.1186/s12866-019-1685-2

**Published:** 2019-12-30

**Authors:** Madina Kozhieva, Natalia Naumova, Tatiana Alikina, Alexey Boyko, Valentin Vlassov, Marsel R. Kabilov

**Affiliations:** 1 Department of Neurology, Neurosurgery and Medical Genetics of the Pirogov Medical University, Ostrovitianova 1, 117513 Moscow, Russia; 2 Institute of Chemical Biology and Fundamental Medicine SB RAS, Lavrentiev 8, Novosibirsk, 630090 Russia; 3 Department of Neuroimmunology of the Federal Center of CVPI, Ostrovitianova 1 str 10, 117513 Moscow, Russia

**Keywords:** Multiple sclerosis, Primary progressive course, Humans, Faecal bacterial assemblage, 16S rRNA gene amplicon sequencing

## Abstract

**Background:**

Gut microbiota has been increasingly acknowledged to shape significantly human health, contributing to various autoimmune diseases, both intestinal and non-intestinal, including multiple sclerosis (MS). Gut microbiota studies in patients with relapsing remitting MS strongly suggested its possible role in immunoregulation; however, the profile and potential of gut microbiota involvement in patients with primary progressive MS (PPMS) patients has received much less attention due to the rarity of this disease form. We compared the composition and structure of faecal bacterial assemblage using Illumina MiSeq sequencing of V3-V4 hypervariable region of 16S rRNA genes amplicons in patients with primary progressive MS and in the healthy controls.

**Results:**

Over all samples 12 bacterial phyla were identified, containing 21 classes, 25 orders, 54 families, 174 genera and 1256 operational taxonomic units (OTUs). The *Firmicutes* phylum was found to be ultimately dominating both in OTUs richness (68% of the total bacterial OTU number) and in abundance (71% of the total number of sequence reads), followed by *Bacteroidetes* (12 and 16%, resp.) and *Actinobacteria* (7 and 6%, resp.). Summarily in all samples the number of dominant OTUs, i.e. OTUs with ≥1% relative abundance, was 13, representing much less taxonomic richness (three phyla, three classes, four orders, six families and twelve genera) as compared to the total list of identified OTUs and accounting for 30% of the sequence reads number in the healthy cohort and for 23% in the PPMS cohort. Human faecal bacterial diversity profiles were found to differ between PPMS and healthy cohorts at different taxonomic levels in minor or rare taxa. Marked PPMS-associated increase was found in the relative abundance of two dominant OTUs (*Gemmiger sp.* and an unclassified *Ruminococcaceae*)*.* The MS-related differences were also found at the level of minor and rare OTUs (101 OTUs). These changes in OTUs’ abundance translated into increased bacterial assemblage diversity in patients.

**Conclusion:**

The findings are important for constructing a more detailed global picture of the primary progressive MS-associated gut microbiota, contributing to better understanding of the disease pathogenesis.

## Background

Identification of specific microorganisms that live in the gut, which was made possible by methodological and instruments’ advances, has contributed to revealing the complex relationship between the microbiota and the host.

Gut microbiota has been increasingly and explicitly acknowledged to shape significantly human health, in particular contributing to various autoimmune diseases, both intestinal and non-intestinal, such as multiple sclerosis, type-1 diabetes, systemic lupus erythematosus, psoriasis, schizophrenia, and some other disorders [1, 2].

Multiple sclerosis (MS), affecting the central nervous system, has an unclear etiology involving both genetic and extrinsic factors. Recent evidence indicates that autoimmune activation may happen in the intestine, following an interaction of bacterial components of the gut flora with local CNS autoreactive T cells [3]. Although by now it is commonly acknowledged that MS patients have dysbiosis compared to healthy individuals [4, 5], the cause-effect relationship between MS and gut microbiota dysbiosis has not been so far unequivocally established [2, 6]. However, some recently reported results are quite suggestive: for instance, transplantation of gut microbiota from multiple sclerosis patients was found to enable spontaneous autoimmune encephalomyelitis in mice [7], and new hypotheses, based on microbes’ involvement, have been proposed as causes for a range of chronic inflammatory diseases, including MS [8]. Accordingly, it has been hypothesized that intervention of the gut microbiome could result in safer novel therapeutic strategies to treat the disease [4, 9]. Yet the development of such strategies needs a more detailed picture of microbiome specifics in the MS-afflicted cohorts in different regions of the world and an improved understanding of the interactions between the microbiota and the host [10].

Pathological and clinical symptoms of MS vary widely [11], the heterogeneity often confusing for diagnostics [12, 13]. Generally several subtypes of the disease are distinguished, among them relapsing-remitting and primary progressive being the most common and the most rare ones, respectively [14]. The former is subdivided into several forms, most commonly with and without relapses, and the latter also is commonly subdivided into the primary and secondary course. There is lack of understanding of pathogenic mechanisms driving progressive MS [15]. In primary progressive MS (PPMS) neurodegenerative mechanisms are believed to dominate, while in the more frequent relapsing forms the autoimmune inflammation is believed to be the major driver, most likely due to different genetic background [16]. Gut microbiota in MS patients has been studied mainly in patients with relapsing remitting MS, and its possible role in immunoregulation was suggested, while the profile and potential in PPMS patients has not been studied yet.

The aim of this study was to investigate gut 16S microbiome of patients with primary progressive multiple sclerosis (PPMS) in comparison with the healthy subjects and to reveal the MS-related shifts.

## Results

After quality filtering and chimera removal a total of 1256 different OTUs were identified at 97% sequence identity level, of which the overwhelming majority (1252) was *Bacteria*, the rest four representing the *Euryarchaeota* phylum of the *Archaea* domain.

Over all samples 12 bacterial phyla were identified, containing 21 classes, 25 orders, 54 families and 174 genera, alongside with unidentified taxa. Most of the bacterial OTUs represented the *Firmcutes* phylum (857 OTUs, or ca. 68% of the total bacterial OTU number), with *Bacteroidetes* and *Actinobacteria* being the second and the third most OTU-rich phyla with 148 (12%) and 84 OTUs (7%), respectively.

Thus the overwhelming majority of OTUs in the faecal bacterial assemblages were ascribed to the *Firmicutes* phylum.

*Clostridia* was the OTU-richest class (669 OTUs), accounting for 53% of the total OTU richness, with *Bacteroidia* (135 OTUs) and *Actinobacteria* (79) contributing 11 and 6%, respectively. Thus these three classes drastically prevailed in faecal bacterial assemblages.

Summarily in all samples the number of dominant OTUs, i.e. OTUs contributing ≥1% into the total sequence number in a sample, was 13, i.e. 1.0% of the total number of OTUs. They represented 3 phyla, 3 classes, 4 orders, 6 families and 12 genera, i.e. much less taxonomic richness as compared to the total list of identified OTUs. The dominant OTUs accounted for 30% of the sequence reads in the healthy cohort and for 23% number in the PPMS cohort.

**Fig. 1:**
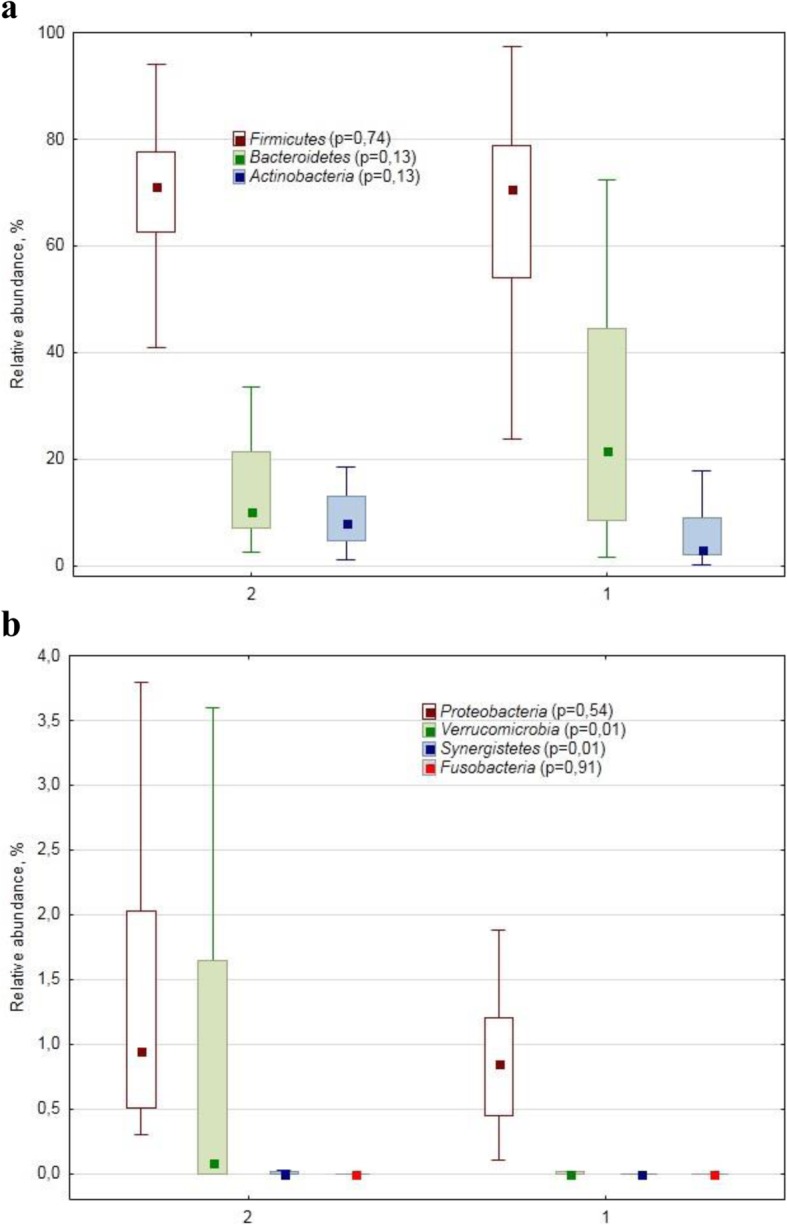
Relative abundance (%) of dominant (**a**) and minor/rare (**b**) phylum-specific 16S rRNA gene amplicon sequences in human faecal samples collected from patients with primary progressive multiple sclerosis (2) and from healthy subjects (1). The marker shows a median; the box shows the 75–25% quartile range, while the lines indicate the fluctuation range. The *p*-values of the Mann-Whitney test for the cohorts’ comparison are shown in brackets after the phylum name

The dominant phyla structure was quite similar in these cohorts (Fig. 1a). However, at the minor/rare phylum level, i.e. phyla with the number of sequence reads contributing less than 1% into the total number of sequence reads, there was a difference in bacterial assemblage structure as *Verrucomicrobiae* sequences were more abundant in PPMS (0.09%) as compared with the healthy (0.00%) cohort (Fig. 1b).

**Fig. 2:**
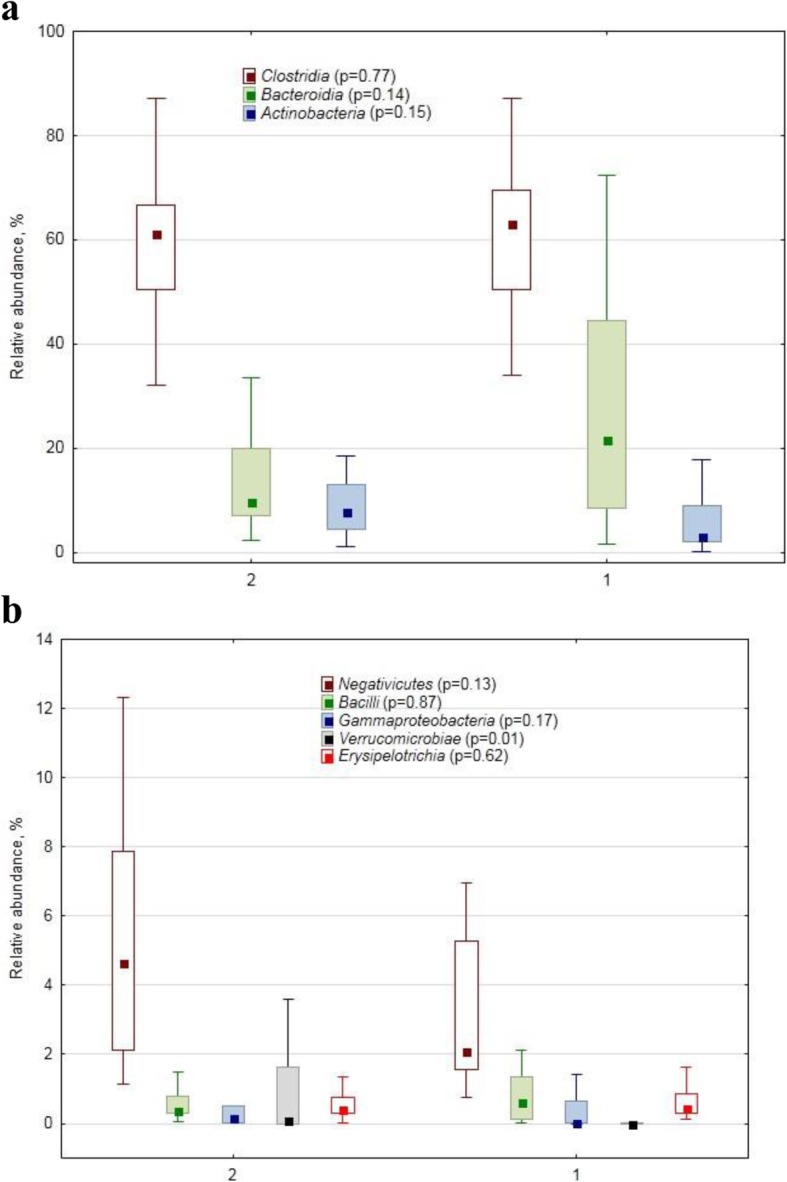
Relative abundance (%) of dominant (**a**) and minor/rare (**b**) class–specific 16S rRNA gene amplicon sequences in human faecal samples collected from healthy subjects (1) and from patients with primary progressive multiple sclerosis (2). The marker shows a median; the box shows the 75–25% quartile range, while the lines indicate the fluctuation range The *p*-values of the Mann-Whitney test for the conhorts’ comparison are shown in brackets after the class name

At the class level the relative abundance of *Clostridia* representatives was lower in the PPMS assemblages (61.2 vs. 63.2%, resp., Fig. 2a). There was also a difference (*p* = 0.0008) between the healthy (0.06%) and PPMS (0.40%) cohorts in the relative abundance of *Deltaproteobacteria* class-specific reads, as well as in *Verrucomicrobiae* (0.00 vs. 0.09%, resp., *p* = 0.01).

**Fig. 3:**
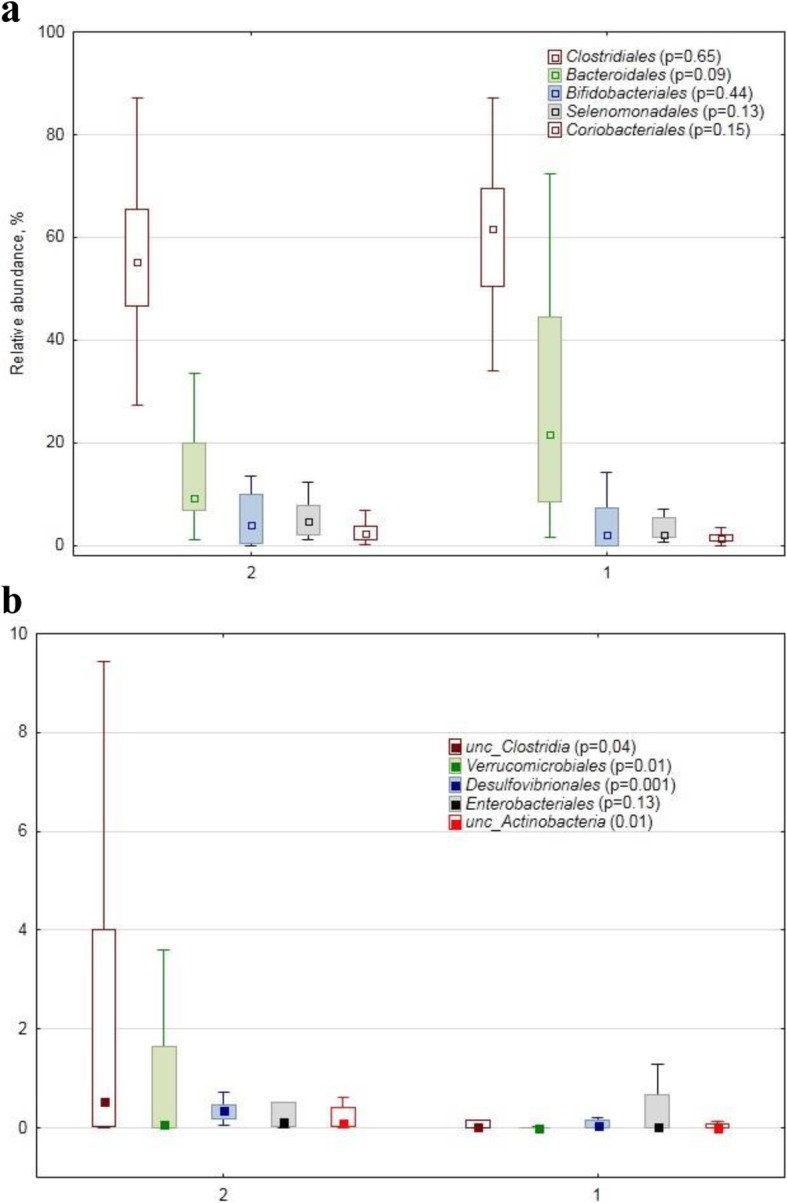
Relative abundance (%) of dominant (**a**) and minor/rare (**b**) order–specific 16S rDNA sequences in human faecal samples collected from the healthy subjects (1) and from the patients with primary progressive multiple sclerosis (2). The marker shows a median; the box shows the 75%25% quartile range, while the lines indicate the fluctuation range The *p*-values of the MannWhitney test for the cohorts’ comparison are shown in brackets after the order name

At the order level no differences were detected among the dominant ones (Fig. 3a), while statistically significant differences between the healthy and PPMS cohorts were found in some minor and rare orders (Fig. 3b): *Actinomycetales* (0.01 vs. 0.03%), *Verrucomicrobiales* (0.00 vs. 0.09, *p* = 0.01), *Desulfovibrionales* (0.06 vs. 0.36%, *p* = 0.001).

At the family level the assemblage structure (Table 1) displayed some differences between healthy and PPMS patients, related mostly to minor or rare families (8 families were explicitly classified, and two were unclassified).

**Table 1:** Relative abundance (%, median values) of family-specific 16S rRNA gene amplicon sequences in human faecal samples collected from healthy subjects and patients with primary progressive multiple sclerosis (PPMS)

Family	PPMS	Healthy	p-value
*Ruminococcaceae*	31.9	28.2	0.23
*Lachnospiraceae*	17.1	26.5	0.06
*Bacteroidaceae*	2.7	1.6	0.84
*Prevotellaceae*	1.8	1.0	0.74
*Bifidobacteriaceae*	4.0	2.1	0.44
*unc. Clostridiales* ^a^	4.2	2.6	0.09
*Coriobacteriaceae*	2.2	1.4	0.15
***unc. Firmicutes***	**2.3**	**0.3**	**0.01**^**b**^
*Veillonellaceae*	2.2	1.9	0.87
***Acidaminococcaceae***	**1.1**	**0.02**	**0.04**
*Porphyromonadaceae*	0.9	0.5	0.23
***unc. Clostridia***	**0.5**	**0.07**	**0.04**
***Eubacteriaceae***	**1.5**	**0.5**	**0.01**
*Rikenellaceae*	0.6	0.2	0.07
*Streptococcaceae*	0.3	0.6	0.59
*Erysipelotrichaceae*	0.4	0.4	0.62
***Verrucomicrobiaceae***	**0.09**	**0.00**	**0.01**
*unc. Bacteria*	0.31	0.06	0.16
*Peptostreptococcaceae*	0.5	0.1	0.12
*Lactobacillaceae*	0.01	0.00	0.10
*Methanobacteriaceae*	0.07	0.00	0.06
*Clostridiaceae_1*	0.01	0.1	0.57
*Sutterellaceae*	0.09	0.06	0.93
***Desulfovibrionaceae***	**0.36**	**0.05**	**0.00**
*Enterobacteriaceae*	0.1	0.3	0.13
***Actinomycetaceae***	**0.03**	**0.01**	**0.01**
*Clostridiales_i.s._XIII*	0.003	0.000	0.17
***Oxalobacteraceae***	**0.004**	**0.000**	**0.03**
***Christensenellaceae***	**0.005**	**0.000**	**0.01**
***Corynebacteriaceae***	**0.003**	**0.000**	**0.01**

^a^ “unc.” stands for unclassified;

^b^ the rows with statistically significant (*P* ≤ 0.05) differences between the cohorts’ medians are highlighted in bold

The *Acidaminococcaceae* family in our study was represented by 3 genera (*Phascolarctobacterium, Acidaminococcus* and some unidentified one) and 8 OTUs, namely *Phascolarctobacterium faecium, Phascolarctobacterium succinatutens, Acidaminococcus intestini, Acidaminococcus fermentans*, as well as 3 unidentified ones. *Eubacteriaceae* family was represented by 2 genera (*Eubacterium, Anaerofustis*) and 9 OTUs; *Verrucomicrobiaceae* had one genus (*Akkermansia*) and one OTU, *Desulfovibrionaceae* was represented by two genera, i.e. *Desulfovibrio* with 7 OTUs, and *Bilophila* with one. *Actinomycetaceae* had 4 genera, with *Actinomyces* contributing 9 OTUs *and Mobiluncus, Varibaculum* and *Trueperella* each contributing just one. *Corynebacteriaceae* had 4 OTUs of the *Corynebacterium* genus. Only one OTU represented *Oxalobacteraceae,* and two OTUs represented *Christensenellaceae family.*

As for the dominant OTUs, we found some marked PPMS-associated increase in the relative abundance of *Gemmiger sp.* and an unclassified *Ruminococcaceae* (Table 2)*.* In contrast to the dominant OTUs, a larger number of minor and rare OTUs were found to show PPMS-related differences: 101 OTUs at the significance level of 0.05 and 85 OTUs at the significance level of 0.10. (Additional file 1: Tables S1 and S2).

**Table 2:** Relative abundance (%, median values) of dominant OTUs in human faecal samples collected from the healthy subjects and patients with primary progressive multiple sclerosis (PPMS)

Dominant OTUs		PPMS	Healthy subjects	p-value
*1*	*Faecalibacterium prausnitzii*	11.2	11.3	0.35
*2*	*Bifidobacterium sp.*	1.8	0.1	0.12
*3*	*unc. Lachnospiraceae* ^a^	2.5	4.2	0.19
*4*	*Ruminococcus bromii*	3.5	2.3	0.22
***5***	***Gemmiger sp.***	**2.2**	**1.0**	**0.03**^**b**^
*6*	*Collinsella sp.*	1.0	0.8	0.47
*7*	*Bacteroides sp.*	1.4	0.7	0.71
*8*	*Blautia wexlerae*	0.4	1.2	0.10
*9*	*Prevotella copri*	1.3	0.0	0.46
*10*	*Eubacterium hallii*	1.8	0.8	0.14
***11***	***unc.Ruminococcaceae***	**1.7**	**0.2**	**0.00**
*13*	*Anaerostipes sp.*	1.1	1.8	0.22
*16*	*Blautia luti*	0.4	1.4	0.08

^a^ “unc.” stands for unclassified;

^b^ the rows with statistically significant (*P* ≤ 0.05) differences between the cohorts’ medians are highlighted in bold

Statistically significant differences between the studied healthy and PPMS cohorts were detected in such indices as OTU richness, Berger-Parker, Flyvbjerg and Mirror (Table 3), all indicating higher diversity in PPMS-associated bacterial assemblage.

**Table 3:** Alpha-biodiversity indices of bacterial assemblages in human faecal samples collected from the healthy subjects and patients with primary progressive multiple sclerosis (PPMS)

Index	PPMS	Healthy	p-value
**Richness**^**a**^	**163**	**129**	**0.03**
Chao1	231	177	0.10
**Berger-Parker**	**0.13**	**0.16**	**0.03**
Simpson	0.95	0.94	0.11
Dominance	0.05	0.06	0.11
Buzas-Gibson	0.020	0.015	0.12
Equitability	0.75	0.73	0.22
Jost	28	22	0.11
Shannon	3.8	3.6	0.14
Robbins	0.35	0.31	0.12
**Flyvbjerg**	**165**	**117**	**0.05**
**Mirror**	**272**	**219**	**0.04**

^a^ Rows with significant (*P* ≤ 0.05) differences between the values are highlighted in bold

## Discussion

We could not compare our results with other PPMS cohorts, as, similar to other researchers [17], we failed to find the data on PPMS-associated gut microbiome obtained using metagenomic methodology. It might seem surprising, as bibliography search commonly produces review articles stating that patients with MS have altered microbiome as compared to healthy people. However, most of such statements pertain to the patients with relapse-remitting subtype of MS as it is the most common subtype of the disease, while PPMS one is rather rare [14] and, consequently, the information on gut bacterial assemblage structure and composition is also scarce.

We found altered gut bacterial assemblage in the PPMS patients as compared with the healthy subjects. At the phylum level no difference in faecal bacterial assemblage structure between the healthy and PPMS cohorts was found in the dominant phyla abundance. However, some PPMS-associated differences were detected in the relative abundance of the rare phyla, i.e. phyla represented by just few OTUs and contributing much less than 1% into the total number of sequences: for example, *Verrucomicrobiae* with just 4 OTUs showed PPMS-related increase due to *Akkermansia muciniphila*. This result agrees with  the earlier finding that increased *A. muciniphila* relative abundance was associated with experimental autoimmune encephalomyelitis (an animal model of MS) [18, 19]. *Akkermansia muciniphila* is a mucin-degrading bacterium, in such capacity not being beneficial for human health; however, it is also a propionogenic bacterium, believed to have several health benefits in humans [20].

*Synergistetes* phylum, with its practically negligible relative abundance and represented by just 6 OTUs, also had increased PPMS-associated relative abundance. The phylum was found to be positively correlated with normal immune homeostasis [21]; however, earlier research found the phylum association with chronic osteomyelitis of the jaw [22]. Albeit the interpretation of the phylum significance, if any, in MS-associated microbiome is difficult, such rare phyla might be important in ecological, physiological and/or pathogenic interplay within the human gut microbiome and between the latter and the host organism.

*Firmicutes* phylum predominated in all samples; and most pronounced MS-related alterations were found also in the relative abundance of some phylum representatives, such as *Acidaminococcaceae, Eubacteriaceae, Christensenellaceae*, as well as some unclassified *Firmicutes* and *Clostridia*. The *Acidaminococcus* and *Phascolarctobacterium* genera, representing *Acidaminococcaceae*, are known as common commensals in the human gut [23, 24], beneficial for health. Both *Eubacteriaceae* genera, detected in our study, namely *Eubacterium* and *Anaerofustis* with 6 and 3 OTUs, respectively, are beneficial gut bacteria [25–27]. Quite a lot of OTUs with differential relative abundance in PPMS and healthy cohorts also belonged to *Firmicutes* phylum.

The increased relative abundance of sequences, representing beneficial bacteria, in PPMS-associated assemblage confuses the pathophysiological interpretation of their association with MS, the situation complies with earlier conclusion that so far little consistency in the MS-associated bacterial taxa has been found [28].

*Desulfovibrionales* order, represented by *Desulfovibrionaceae* family with *Bilophila* and *Desulfovibtio* genera, in their turn represented respectively by one OTU (*Bilophila wadsworthia*) and 7 OTUs of unclassified *Desulfovibrio*, were found to be more abundant in the PPMS cohort. *Bilophila* is a known pathobiont [29, 30]. Although *Desulfovibrio piger* is found in some healthy human guts, a greater abundance of this species may be associated with certain gastrointestinal diseases, such as inflammatory bowel disease [31] or autism [32].

Biodiversity indices serve to compact information about communities, assemblages, guilds etc. of living organisms; thus the indices are useful for comparing large arrays of metagenomic data. We found that four α-biodiversity indices were higher in the primary progressive MS cohort, indicating the presence of a bigger set of players, albeit minor and rare ones, in the PPMS-associated gut microbiota as compared with the healthy one. However, earlier a trend towards lower species richness was found in relapsing-remitting MS patients with active disease as compared with the healthy controls [33], using the same methodology, i.e.V3-V4 rRNA gene region for PCR amplification and sequencing by Illumina MiSeq, as in our study.

The aberrant MS-related gut microbiota, found in our study, supports the idea of using diet and/or other means to modulate gut microbiota in an attempt to alleviate [9] and possibly prevent MS [34] even without certainty about cause-effect relationship between microbiome members and MS progress. However, the rarity of most of the PPMS-associated gut bacteria makes further investigation of their physiological and pathogenic relevance quite challenging.

All the PPMS patients included in our study received neither oral nor injective disease modifying therapies (DMTs), which could have modified the gut microbiome, as was shown earlier for patients with the relapsing forms of MS [35]. Quite recently gut microbiota-dependent T cells were found altered in secondary progressive multiple sclerosis [36], suggesting similar possibility in the PPMS as well.

## Conclusions

To our knowledge, this study presents the first inventory of the faecal bacterial assemblage composition and structure in patients affected by primary progressive multiple sclerosis. We provide evidence that human faecal bacterial diversity profiles revealed differences between PPMS and healthy cohorts at different taxonomic levels. A number of MS-associated changes, detected in some rare bacterial OTUs’ abundance, translated into increased diversity in MS patients. The findings are important to get a more detailed global picture of the MS-associated bacterial assemblage, contributing to better understanding of the disease pathogenesis (associated both with immunoregulation and neurodegeneration) and suggesting, at least for the alleviation therapy, possible avenues.

## Methods

### Participants and faecal sample collection

Healthy subjects (*n* = 15) and patients with PPMS (*n* = 15) as diagnosed by MacDonald criteria [37] were recruited for the trial. Demographic characteristics of these patients are in Table 4. All patients underwent clinical examination to assess their neurological status and disability according to the Expanded Disability Status Scale (EDSS) [38]. Median of MS duration was 3.6 years (2.0–5.0). All included MS patients had PPMS with confirmed EDSS progression at least for 12 months, according to generally accepted criteria [16], which are validated in Russia [39]. None of the PPMS patients received any DMTs or oral or intravenous courses of corticosteroids. All patients were duly informed and gave their consent to the study and signed the Information Consent.

**Table 4:** Demographics of the study cohorts (medians)

Property	Healthy (*n* = 15)	PPMS (*n* = 15)
Age, years (range)	23 a^¥^ (20–73)	45 a (25–56)
Females, %	44	40
Males, %	56	60
BMI (range)	24 a (17–30)	22 a (20–27)

^¥^The values in rows followed by the same letters do not differ significantly at *P* ≤ 0.05 level

Faecal samples were collected into 10 ml sterile faecal specimen containers and stored frozen at approximately −20 °C. Samples were transferred to the laboratory within 1 week of collection and stored at − 80 °C until used for DNA extraction. The samples were collected at least one month prior to corticosteroid treatment.

The protocol of the study was approved by the Ethic Committee of the Pirogov National Science and Research Medical University. All clinical aspects of the study were supervised by a neurologist. New medicines, sorbents and/or laxatives (including magnesium salts and castor oil), as well as any diet changes, were cancelled or not started at least one week prior to faecal samples collection.

### DNA extraction and sequencing

DNA was extracted from 50 to 100 mg of thawed patient faecal samples using MetaHIT protocol [40]. The bead-beating was performed using TissueLyser II (Qiagen, Germany), for 10 min at 30 Hz and 0.75 ml Zirconia/Silica Beads 0.1 mm (BioSpec Products). The quality and quantity were analysed by Nanodrop-1000 (ThermoScientific) and Qubit (Invitrogen) respectively.

The 16S rRNA gene region was amplified with the primer pair V3-V4 combined with Illumina adapter sequences [41]. PCR amplification was performed as described earlier [42]. All PCR reactions used 25 ng of faecal DNA as template and were performed in triplicates for each sample. Then the triplicates were pooled, and a total of 200 ng PCR product for each sample was pooled together and purified through MinElute Gel Extraction Kit (Qiagen, Germany). The obtained libraries were sequenced with 2 × 300 bp paired-ends reagents on MiSeq (Illumina, USA) in SB RAS Genomics Core Facility (ICBFM SB RAS, Novosibirsk, Russia). The read data were deposited in GenBank under the study accession PRJNA565173 and the sample accession SRP221464.

### Bioinformatic and statistical analyses

Raw sequences were analyzed with UPARSE pipeline [43] using Usearch v.11.0. The UPARSE pipeline included merging of paired reads; read quality filtering; length trimming; merging of identical reads (dereplication); discarding singleton reads; removing chimeras and operational taxonomic unit (OTU) clustering using the UPARSE-OTU algorithm. The OTU sequences were assigned a taxonomy using the SINTAX [44] and 16S RDP training set v.16 [45].

Taxonomic structure of thus obtained sequence assemblages, i.e. a collection of different species at one site at one time, was estimated by the ratio of the number of taxon-specific sequence reads to the total number of sequence reads, i.e. by the relative abundance of taxa, expressed as percentage.

Comparison of relative abundances of different bacterial taxa in faecal samples of the control and PPMS group was carried out by the Mann-Whitney nonparametric test for independent samples using the Statistica v.13.3 software (Statsoft, USA). The α-biodiversity indices were calculated using Usearch. All data are presented as median values.

## Supplementary information

**Additional file 1: Tables S1** and **S2** with relative abundance (%) of OTUs in human faecal samples collected from healthy subjects and patients with primary progressive multiple sclerosis.

## Data Availability

The read data were deposited in GenBank under the study accession PRJNA565173 and the sample accession SRP221464.

## Abbreviations

DMT: Disease modifying therapy
EDSS: Expanded Disability Status Scale
MS: Multiple sclerosis
OTU: Operational taxonomic unit
PPMS: Primary progressive multiple sclerosis
