# Supplementary material for: Primary progressive multiple sclerosis in a Russian cohort: relationship with gut bacterial diversity
Source: BMC Microbiol. 2019 Dec 30;19:309. doi: 10.1186/s12866-019-1685-2 (PMC6937728; doi:10.1186/s12866-019-1685-2)
Supplement: Supplementary file 1 — Additional file 1: Tables S1 and S2 with relative abundance (%) of OTUs in human faecal samples collected from healthy subjects and patients with primary progressive multiple sclerosis. [file 12866_2019_1685_MOESM1_ESM.doc]

**Additional file 1**

Table S1. Relative abundance (%) of OTUs in human faecal samples collected from healthy subjects and patients with primary progressive multiple sclerosis (PPMS); only OTUs with differential p-values less or equal to 0.05 are shown in the table.

| **OTU** | **OTU’s name** | **PPMS** | **Healthy** | **p-value** |
| --- | --- | --- | --- | --- |
| 5 | *unc.* Gemmiger* | 2.22 | 0.98 | 0.03 |
| 11 | *unc. Ruminococcaceae* | 1.70 | 0.23 | 0.00 |
| 21 | *Fusicatenibacter saccharivorans* | 0.10 | 0.41 | 0.05 |
| 27 | *unc. Clostridia* | 0.12 | 0.00 | 0.02 |
| 30 | *Akkermansia muciniphila* | 0.09 | 0.00 | 0.01 |
| 35 | *unc. Ruminococcaceae* | 0.56 | 0.10 | 0.00 |
| 49 | *unc. Clostridiales* | 0.09 | 0.00 | 0.02 |
| 55 | *unc. Firmicutes* | 0.19 | 0.00 | 0.01 |
| 57 | *unc. Ruminococcaceae* | 0.27 | 0.03 | 0.01 |
| 60 | *unc. Firmicutes* | 0.52 | 0.10 | 0.05 |
| 61 | *unc. Lachnospiraceae* | 0.09 | 0.25 | 0.05 |
| 73 | *unc. Firmicutes* | 0.01 | 0.00 | 0.03 |
| 95 | *unc. Ruminococcaceae* | 0.01 | 0.00 | 0.05 |
| 102 | *unc. Clostridiales* | 0.08 | 0.01 | 0.01 |
| 110 | *Bilophila. wadsworthia* | 0.15 | 0.01 | 0.00 |
| 115 | *Barnesiella. intestinihominis* | 0.34 | 0.06 | 0.05 |
| 125 | *Odoribacter. splanchnicus* | 0.10 | 0.02 | 0.05 |
| 144 | *unc. Lachnospiraceae* | 0.02 | 0.16 | 0.02 |
| 159 | *unc. Alistipes* | 0.06 | 0.01 | 0.00 |
| 168 | *Eggerthella. lenta* | 0.02 | 0.00 | 0.03 |
| 169 | *unc. Clostridiales* | 0.09 | 0.00 | 0.04 |
| 179 | *unc. Ruminococcaceae* | 0.03 | 0.01 | 0.01 |
| 198 | *Parabacteroides. distasonis* | 0.08 | 0.03 | 0.03 |
| 205 | *unc. Clostridiales* | 0.02 | 0.08 | 0.00 |
| 207 | *unc. Clostridiales* | 0.01 | 0.00 | 0.00 |
| 213 | *unc. Ruminococcaceae* | 0.10 | 0.01 | 0.03 |
| 234 | *unc. Clostridiales* | 0.09 | 0.16 | 0.05 |
| 238 | *unc. Clostridium. IV* | 0.003 | 0.000 | 0.03 |
| 245 | *unc. Ruminococcaceae* | 0.02 | 0.00 | 0.05 |
| 280 | *unc. Ruminococcaceae* | 0.003 | 0.000 | 0.04 |
| 317 | *unc. Bacteria* | 0.01 | 0.00 | 0.00 |
| 319 | *Anaerotruncus. colihominis* | 0.004 | 0.000 | 0.01 |
| 335 | *unc. Streptococcus* | 0.004 | 0.000 | 0.01 |
| 356 | *unc. Porphyromonadaceae* | 0.01 | 0.00 | 0.04 |
| 359 | *unc. Firmicutes* | 0.004 | 0.000 | 0.04 |
| 368 | *unc. Clostridiales* | 0.01 | 0.00 | 0.01 |
| 373 | *unc. Desulfovibrio* | 0.003 | 0.000 | 0.02 |
| 378 | *unc. Clostridiales* | 0.01 | 0.00 | 0.02 |
| 426 | *unc. Ruminococcaceae* | 0.003 | 0.000 | 0.04 |
| 442 | *unc. Ruminococcaceae* | 0.004 | 0.000 | 0.01 |
| 456 | *unc. Firmicutes* | 0.01 | 0.00 | 0.00 |
| 473 | *unc. Clostridiales* | 0.003 | 0.000 | 0.03 |
| 508 | *unc. Clostridia* | 0.003 | 0.000 | 0.00 |
| 512 | *Christensenella. minuta* | 0.003 | 0.000 | 0.01 |
| 525 | *unc. Lachnospiraceae* | 0.004 | 0.000 | 0.04 |
| 526 | *unc. Ruminococcaceae* | 0.01 | 0.00 | 0.04 |
| 531 | *unc. Firmicutes* | 0.003 | 0.000 | 0.04 |
| 556 | *unc. Clostridiales* | 0.003 | 0.000 | 0.00 |
| 577 | *unc. Firmicutes* | 0.000 | 0.004 | 0.02 |
| 606 | *unc. Oxalobacteraceae* | 0.002 | 0.000 | 0.02 |
| 648 | *unc. Anaerofilum* | 0.003 | 0.000 | 0.05 |
| 678 | *unc. Clostridiales* | 0.003 | 0.000 | 0.03 |
| 881 | *unc. Clostridiales* | 0.04 | 0.01 | 0.02 |
| 891 | *unc. Corynebacterium* | 0.00 | 0.00 | 0.02 |
| 954 | *unc. Clostridiales* | 0.00 | 0.03 | 0.01 |
| 1052 | *unc. Blautia* | 0.00 | 0.05 | 0.02 |
| 1071 | *Faecalibacterium. prausnitzii* | 0.01 | 0.03 | 0.04 |
| 1073 | *Clostridium. lactatifermentans* | 0.000 | 0.003 | 0.01 |
| 1130 | *unc. Clostridiales* | 0.00 | 0.01 | 0.02 |
| 1155 | *unc. Lachnospiraceae* | 0.00 | 0.01 | 0.02 |
| 1184 | *unc. Clostridiales* | 0.01 | 0.09 | 0.03 |
| 1225 | *unc. Ruminococcaceae* | 0.003 | 0.000 | 0.01 |
| 1242 | *Faecalibacterium. prausnitzii* | 0.00 | 0.01 | 0.03 |

* “unc.” stands for unclassified.

Table S2. Relative abundance (%) of OTUs in human faecal samples collected from healthy subjects and patients with primary progressive multiple sclerosis (PPMS); only OTUs with differential p-values ranging 0.05-0.10 are shown in the table.

| **OTU** | **OTU’s name** | **PPMS** | **Healthy** | **p-value** |
| --- | --- | --- | --- | --- |
| 8 | *Blautia wexlerae* | 0.42 | 1.19 | 0.097 |
| 16 | *Blautia luti* | 0.36 | 1.39 | 0.081 |
| 22 | *Methanobrevibacter smithii* | 0.07 | 0.00 | 0.067 |
| 29 | *Dorea longicatena* | 0.24 | 0.70 | 0.081 |
| 38 | *unc.* Romboutsia* | 0.26 | 0.07 | 0.097 |
| 65 | *Alistipes onderdonkii* | 0.06 | 0.01 | 0.104 |
| 107 | *Bacteroides massiliensis* | 0.01 | 0.00 | 0.092 |
| 248 | *unc. Lachnospiraceae* | 0.00 | 0.04 | 0.061 |
| 250 | *unc. Enterobacteriaceae* | 0.004 | 0.000 | 0.103 |
| 256 | *unc. Oscillibacter* | 0.003 | 0.000 | 0.101 |
| 278 | *unc. Firmicutes* | 0.05 | 0.00 | 0.086 |
| 333 | *unc. Clostridiales* | 0.01 | 0.00 | 0.064 |
| 336 | *unc. Firmicutes* | 0.03 | 0.00 | 0.063 |
| 345 | *Butyricimonas paravirosa* | 0.01 | 0.00 | 0.092 |
| 391 | *unc. Lachnospiraceae* | 0.003 | 0.000 | 0.076 |
| 437 | *unc. Roseburia* | 0.01 | 0.08 | 0.056 |
| 446 | *unc. Lachnospiraceae* | 0.000 | 0.004 | 0.074 |
| 491 | *unc. Ruminococcaceae* | 0.004 | 0.000 | 0.100 |
| 496 | *Actinomyces turicensis* | 0.01 | 0.00 | 0.069 |
| 649 | *unc. Actinomyces* | 0.004 | 0.000 | 0.088 |
| 670 | *unc. Ruminococcaceae* | 0.025 | 0.007 | 0.100 |
| 709 | *unc. Clostridiales* | 0.003 | 0.000 | 0.068 |
| 786 | *unc. Lachnospiraceae* | 0.00 | 0.01 | 0.067 |
| 825 | *Roseburia intestinalis* | 0.18 | 0.55 | 0.071 |
| 1083 | *unc. Blautia* | 0.00 | 0.03 | 0.073 |
| 1098 | *unc. Lachnospiraceae* | 0.01 | 0.00 | 0.069 |
| 1168 | *unc. Lachnospiraceae* | 0.000 | 0.003 | 0.093 |
| 1186 | *unc. Clostridiales* | 0.000 | 0.004 | 0.071 |
| 1198 | *unc. Lachnospiraceae* | 0.000 | 0.004 | 0.076 |
| 1229 | *unc. Lachnospiraceae* | 0.00 | 0.01 | 0.089 |

** “unc.” stands for unclassified.
